# Supplementary material for: Human multilineage pro-epicardium/foregut organoids support the development of an epicardium/myocardium organoid
Source: Nat Commun. 2022 Nov 15;13:6981. doi: 10.1038/s41467-022-34730-7 (PMC9666429; doi:10.1038/s41467-022-34730-7)
Supplement: Supplementary file 1 — Supplementary Information [file 41467_2022_34730_MOESM1_ESM.pdf]

SUPPLEMENTARY INFORMATION

Human Multilineage Organoids Recapitulate Pro-Epicardium/Septum Transversum/Posterior Foregut and Support the Development of Epicardium-Myocardium Organoids

Mariana A. Branco[1,2], Tiago P. Dias[1,2], Joaquim M.S. Cabral[1,2], Perpetua Pinto-do-Ó[3,4], Maria Margarida Diogo[1,2,*]

[1]iBB – Institute for Bioengineering and Biosciences and Department of Bioengineering, Instituto Superior Técnico, Universidade de Lisboa, Lisboa, Portugal

[2]Associate Laboratory i4HB — Institute for Health and Bioeconomy at Instituto Superior Técnico, Universidade de Lisboa, Lisboa, Portugal

[3]Stem Cells in Regenerative Biology and Repair group, Instituto Nacional de Engenharia Biomédica (INEB), Instituto de Investigação e Inovação em Saúde (i3S), Universidade do Porto, Porto, Portugal

[4]Instituto de Ciências Biomédicas Abel Salazar, Universidade do Porto, Porto, Portugal

*margarida.diogo@tecnico.ulisboa.pt

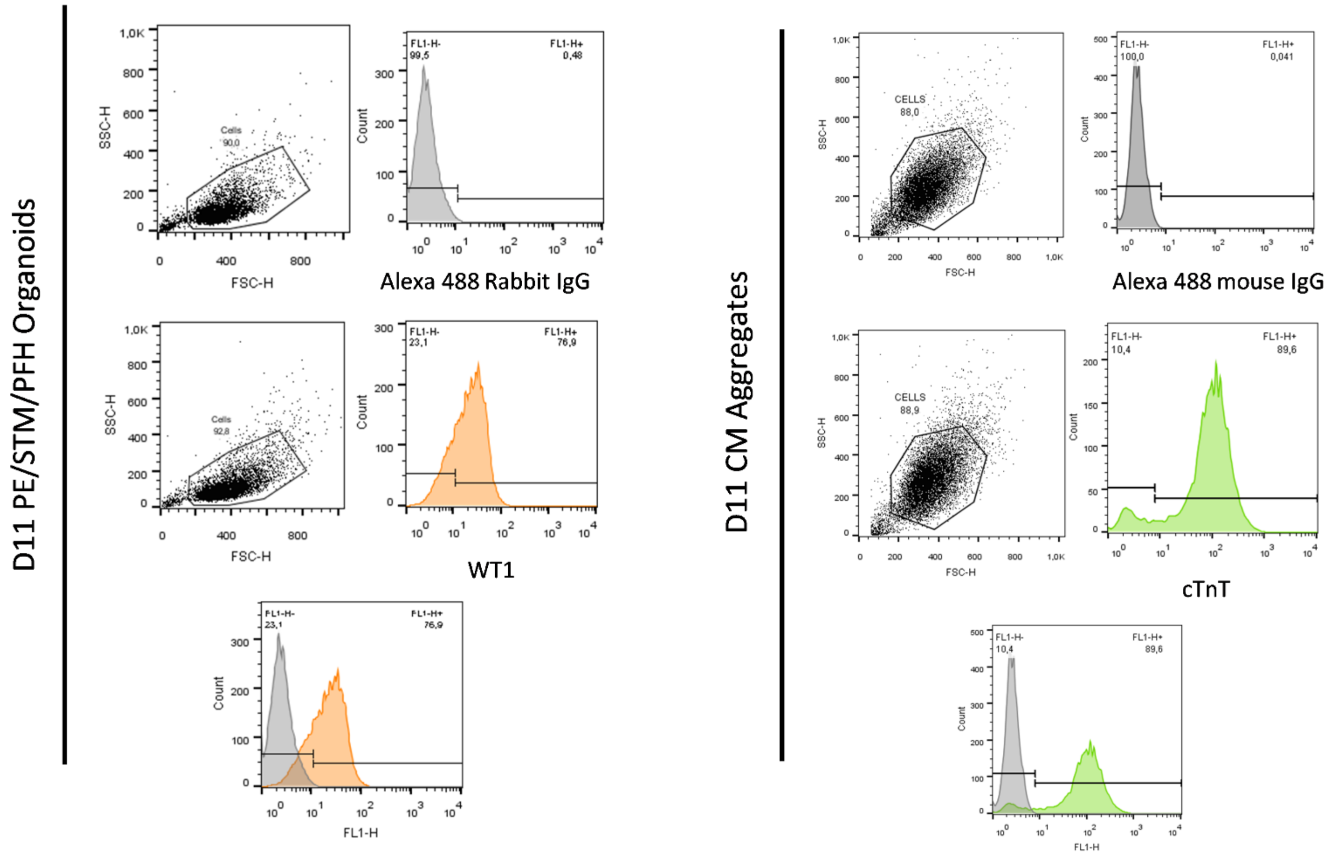

Supplementary Figure 1: Representative gating strategy for cTnT and WT1 positive cell quantification by flow cytometry.

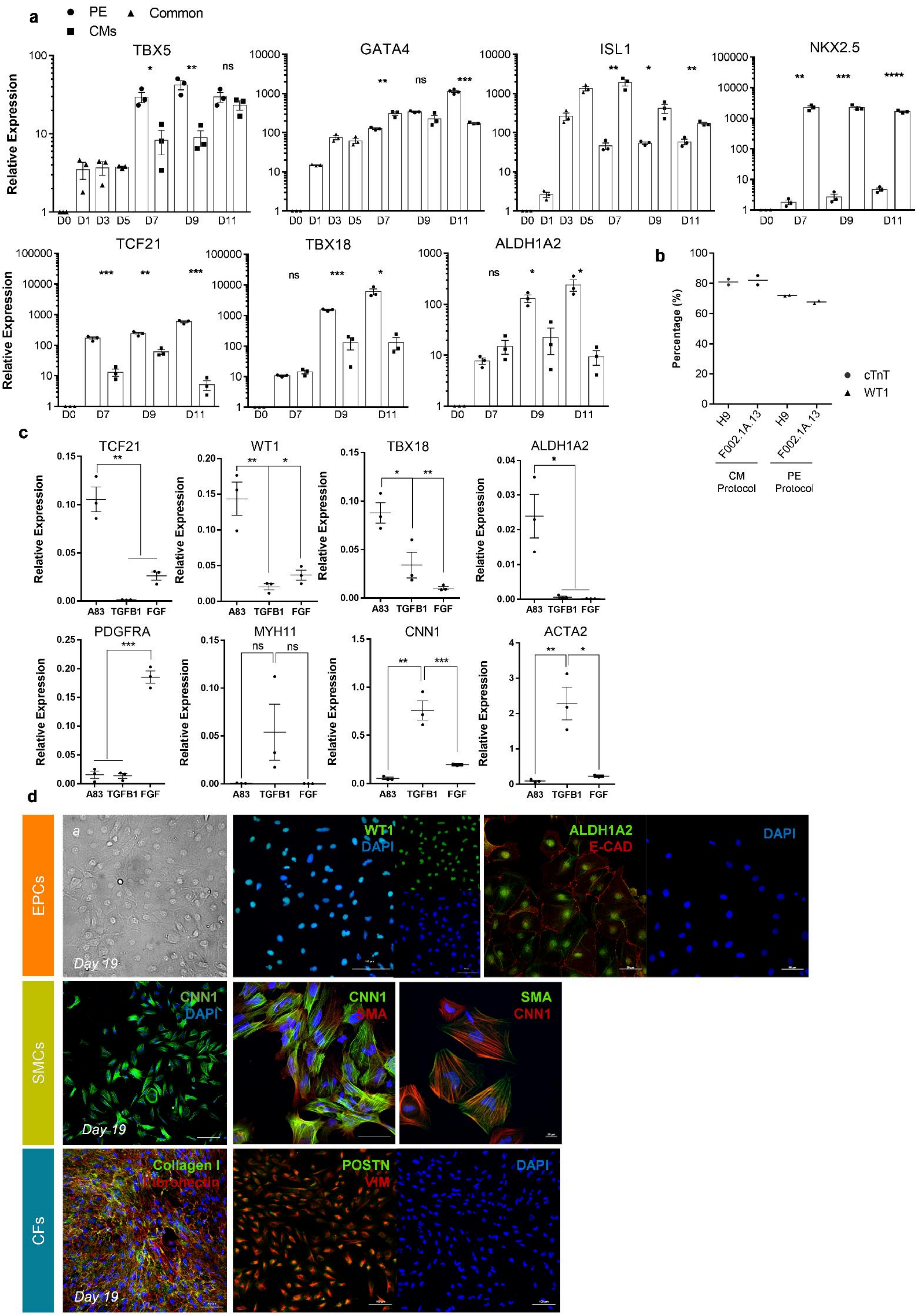
a
PE
CMs
Common
TBX5
GATA4
ISL1
NKX2.5
Relative Expression
D0
D1
D3
D5
D7
D9
D11
TCF21
TBX18
ALDH1A2
b
Percentage (%)
cTnT
WT1
H9
F002.1A.13
CM Protocol
PE Protocol
c
TCF21
WT1
TBX18
ALDH1A2
PDGFRA
MYH11
CNN1
ACTA2
A83
TGFB1
FGF
d
EPCs
SMCs
CFs
Day 19
WT1
DAPI
ALDH1A2
E-CAD
CNN1
SMA
Collagen I
Fibronectin
POSTN
VIM

Supplementary Figure 2: PE and CM differentiation protocols follow the temporal sequential expression profile of key genes involved in PE and CM specification and PE cell showed the capacity to differentiate into SMCs and CFs. a, Expression profile of *TBX5, GATA4, ISL1, NKX2.5, TCF21, ALDH1A2 and TBX18* genes from D0 up to D11 of differentiation for PE and CM differentiation conditions. Values are normalized to *GAPDH* and to D0 of differentiation. Data are represented as mean ± SEM of n=3 independent experiments. Exact p-values: TBX5 D7 p=0.0142, D9 p=0.0055, D11 p=0.2983; GATA4 D7 p=0.0055, D9 p=0.0835, D11 p=0.0001; ISL1 D7 p=0.0065, D9 p=0.0301, D11 p=0.0017; NKX2.5 D7 p=0.0011, D9 p=0.0009, D11 p<0.0001; TCF21 D7 p=0.007, D9 p=0.0022, D11 p=0.0002; TBX18 D7 p=01522, D9 p=0.0001, D11 p=0.0123; ALDH1A2 D7 p=0.1960, D9 p=0.0117, D11 p=0.0191. **b**, Percentage of WT1 and cTnT positive cells after 11 days of differentiation for both PE and CM differentiation protocols, using H9 and F002.1A.13 cell lines. n=2 independent experiments. **c**, Gene expression profile of the epicardial, SMCs and CFs markers after 8 days of EMT differentiation protocols. A83, TGFB1 and FGF columns represents the protocol used to obtain $WT1^{+}$ epicardial-like cells, to differentiate PE aggregates into SMCs and to differentiate PE aggregates into CFs, respectively, after PE replating. Values are normalized to *GAPDH*. Data are represented as mean ± SEM of n=3 independent experiments (exact p-values: CNN1 – A83 vs TGB1 p=0.0023, A83 vs FGF p= 0.0003; ACTA2 - A83 vs TGB1 p=0.0091, A83 vs FGF p= 0.014; PDGFRA – FGF vs A83 p=0.0002, FGF vs TGFB2 p=0.0001; ALDH1A2 – A83 vs TGFB1 p=0.0201, A83 vs FGF p=0.0188; TBX18 – A83 vs TGFB1 p=0.0336, A83 vs FGF p=0.0020; WT1 – A83 vs TGFB1 p=0.0066, A83 vs FGF p=0.0117; TCF21 – A83 vs TGFB1 p=0.0012, A83 vs FGF p=0.0040. **d**, IF staining of replated D19 PE cells, SMCs and CFs. Scale bars, 100 µm. ns, not statistic significant, * $p < 0.05$, ** $p < 0.01$, *** $p < 0.001$ and **** $p < 0.0001$.

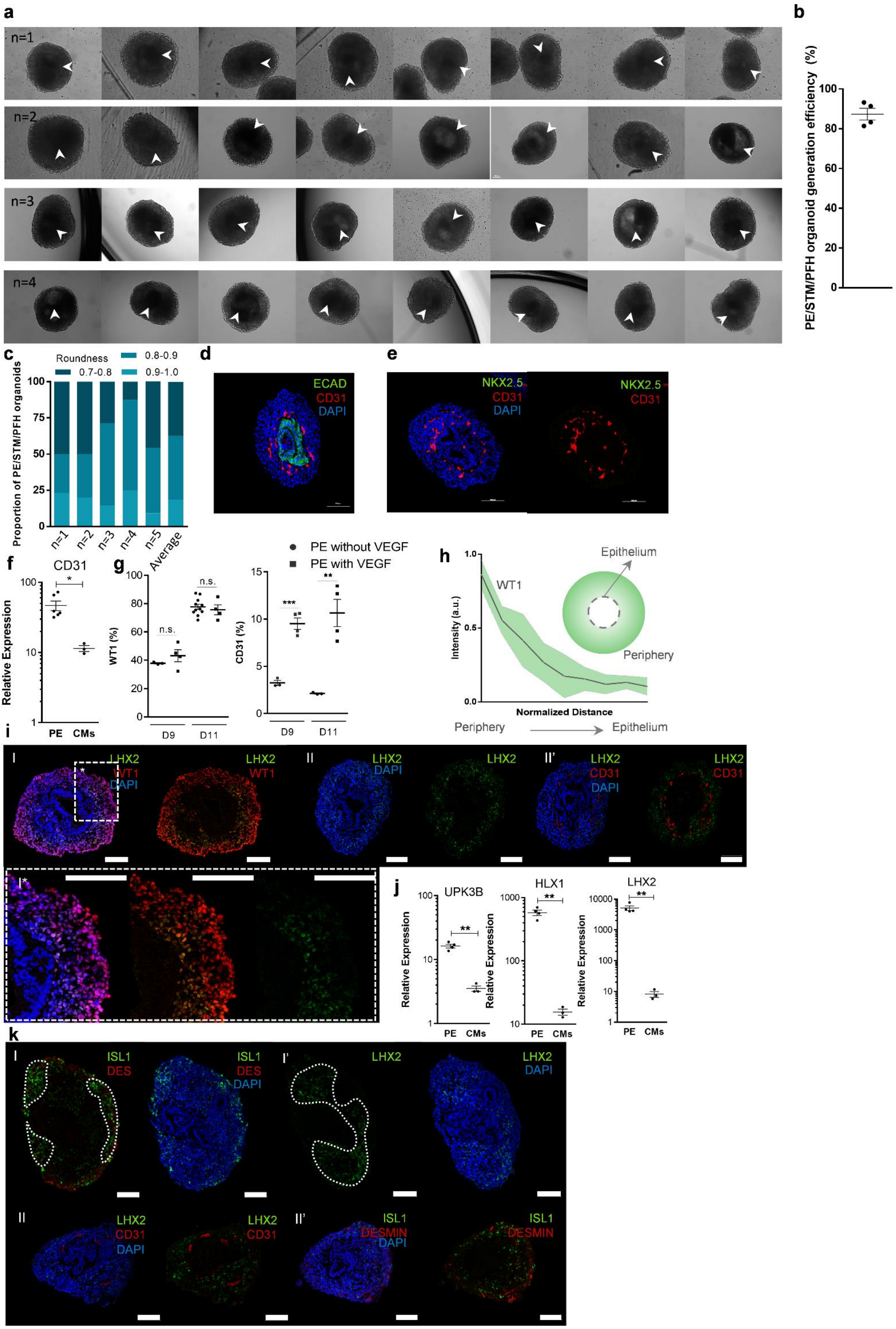
a
n=1
n=2
n=3
n=4
b
PE/STM/PFH organoid generation efficiency (%)
c
Roundness
0.7-0.8
0.8-0.9
0.9-1.0
Proportion of PE/STM/PFH organoids
n=1
n=2
n=3
n=4
n=5
Average
d
ECAD
CD31
DAPI
e
NKX2.5
CD31
DAPI
f
CD31
Relative Expression
PE
CMs
g
WT1 (%)
n.s.
D9
D11
CD31 (%)
PE without VEGF
PE with VEGF
h
WT1
Intensity (a.u.)
Normalized Distance
Epithelium
Periphery
Periphery
Epithelium
i
LHX2
WT1
DAPI
LHX2
CD31
j
UPK3B
HLX1
LHX2
Relative Expression
PE
CMs
k
ISL1
DES
DAPI
LHX2
CD31
DESMIN

Supplementary Figure 3: PE/STM/PFH organoids recapitulate an early stage of pro-epicardium, septum transversum, posterior foregut and hepatic epithelium bud development. **a**, Representative BF images of 8 PE/STM/PFH organoids at D11 of differentiation for n=4 independent biological replicates. White arrows highlight the lumens/epithelial structures. **b**, Efficiency of PE/STM/PFH organoids generation in each independent experiment. Data are represented as mean ± SEM of n=4 independent experiments. **c**, Characterization of PE/STM/PFH organoids roundness, n = 5 independent biological replicates. Data shows the percentage of organoids that present a roundness value between 0.7-0.8; 0.8-0.9 and 0.9-1. **(d-e)**, Representative IF staining of D11 PE/STM/PFH organoid section, highlighting the staining of E-cadherin within the epithelial structure (d) and the absence of NKX2.5 staining in CD31 positive endothelial cells (e). **f**, Gene expression profile of *CD31* at D11 of differentiation for PE and CM differentiation conditions. Values are normalized to *GAPDH* and D0 of differentiation. Data are represented as mean ± SEM of n=3 (CMs) and n=6 (PE) independent experiments. Exact p-value: p=0.0126. **g**, Percentage of WT1 and CD31 positive cells at D9 and D11 of differentiation for both control PE and PE with VEGF supplementation from D7. Data are represented as mean ± SEM of n=3 ($CD31^+$ D9 and D11, $WT1^+$ D9), n=4 ($CD31^+$ and $WT1^+$ D9 VEGF and D11 VEGF) and n=10 ($WT1^+$ D11) independent experiments. Exact p-value: D9 p=0.0004, D11 p=0.0041. **h**, Fluorescence intensity analysis showing expression of WT1 plotted versus the distance from the edge (left) to the epithelium (right) of PE/STM/PFH organoids at D11 of differentiation. Grey traces represent the mean and shaded bars represent the SD (n=9 PE/STM/PFH organoids derived from 3 separate differentiations). **i**, Representative IF staining of D11 PE/STM/PFH organoid sections, highlighting the gradient of WT1 staining and the more pronounced staining of LHX2 towards the epithelial structures. Scale bars, 100 µm. **j**, Gene expression profile of *UPK3B, HLX1 and LHX2* at D11 of differentiation for PE and CM differentiation conditions. Values are normalized to *GAPDH* and D0 of differentiation. Data are represented as mean ± SEM of n=4 (UPK3B, HLX1, LHX2 PE) and n=3 (UPK3B, HLX1, LHX2 CMs) independent experiments. Exact p-values: UPK3B p=0.0026; HLX1 p=0.0097; LHX2 p=0.0044. **k**, Representative IF staining of D11 PE/STM/PFH organoid sections, highlighting the staining of LHX2 and ISL1 subpopulations, which seem to not overlap. The " ' " nomenclature present in IF images was used to refer that the different stainings represent a different slice from the same organoid. Scale bars, 100 µm. ns, not statistic significant, *p< 0.05, **p < 0.01, ***p < 0.001. Source data are provided as a Source Data file.

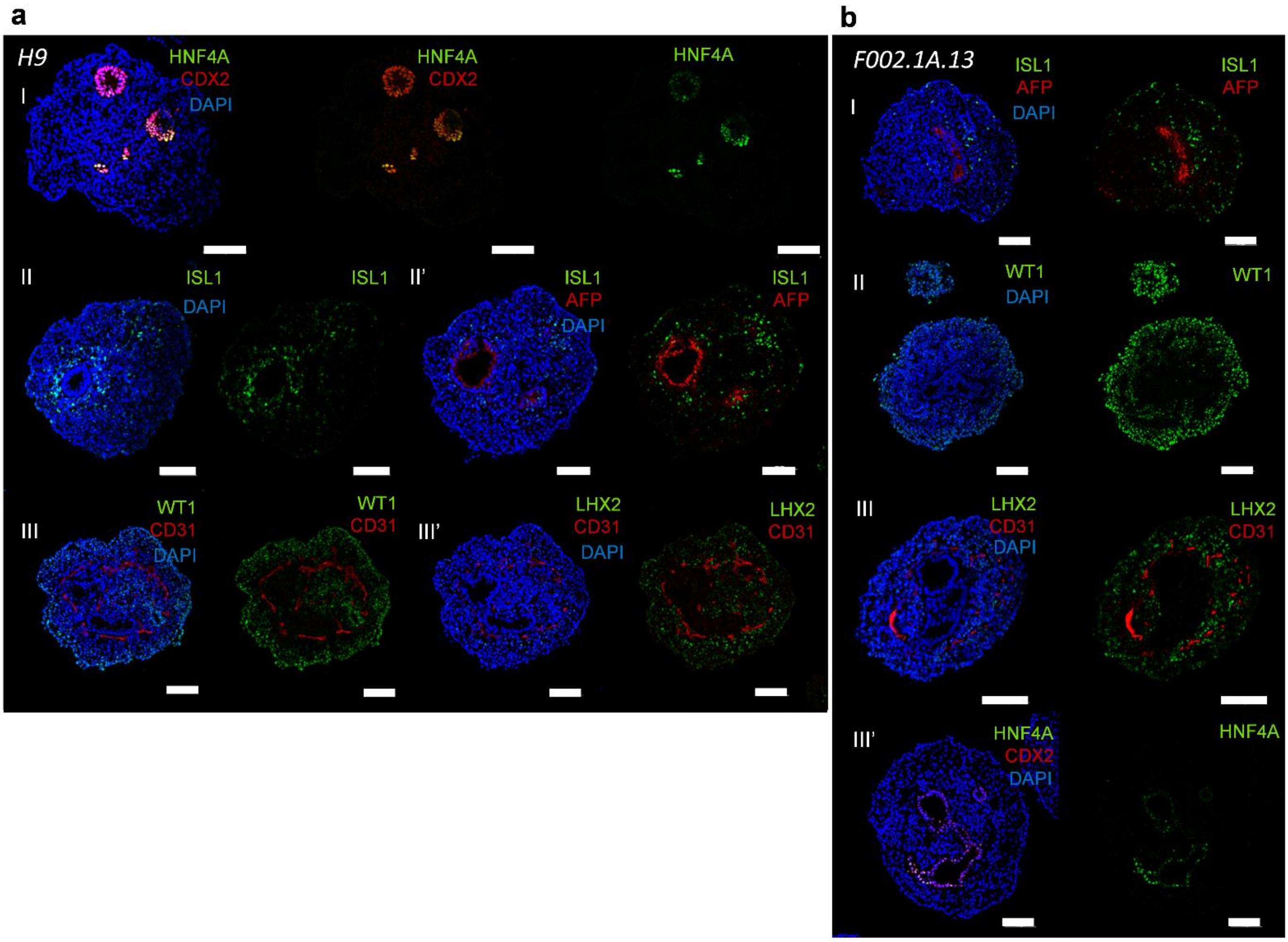

Supplementary Figure 4: PE/STM/PFH organoids were robustly obtained across different hPSC lines. Representative IF staining of D11 PE/STM/PFH organoid sections for H9 and F002.1A.13 hPSC lines, highlighting WT1, LHX2, ISL1, CD31, CDX2, AFP and HNF4A positive cell populations. The " ' " nomenclature present in IF images was used to refer that the different stainings represent a different slice from the same organoid.

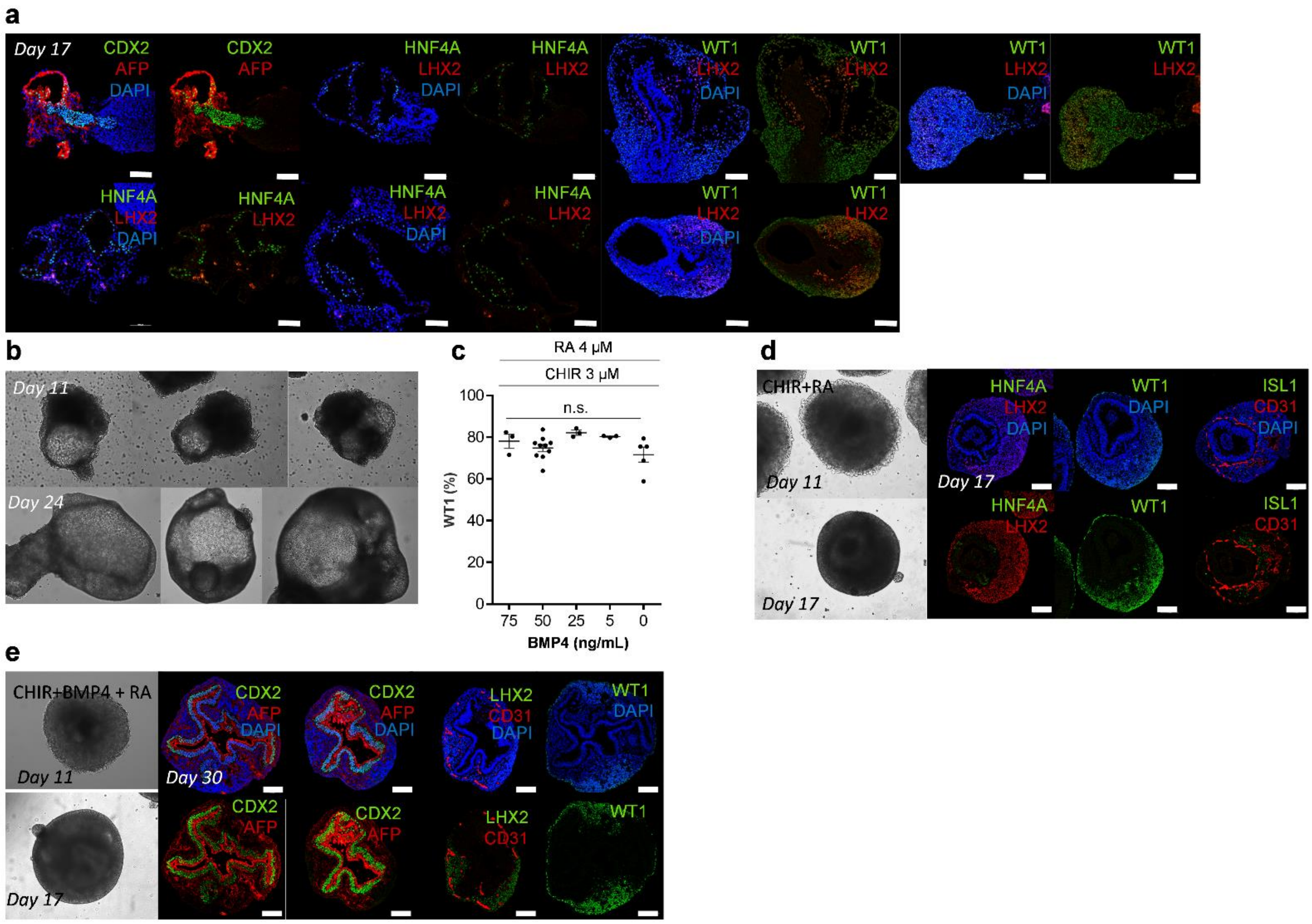

Supplementary Figure 5. WNT and RA are the crucial signals for the development of PE/STM/PFH organoids. a, Representative IF images of D17 organoids obtained from "CHIR" condition, highlighting the cystic hepatoblast-like structures. **b**, Representative BF images of D11 and D24 organoids obtained for the "CHIR+BMP4" condition. **c**, Study the impact of BMP4 concentration variation from 75 – 0 ng/mL from D5 to D7 of differentiation, on the percentage of WT1 positive cells for D11 PE/STM/PFH organoids, assessed by flow cytometry analysis. RA concentration was fixed at 4 µM and CHIR concentration at 3 µM, for all the tested conditions. Data are represented as mean ± SEM of n=3 (75, 25, 5 ng/mL), n=10 (50 ng/mL) and n=5 (0 ng/mL) independent experiments. **d**, Representative BF and IF images of D11 and D17 organoids obtained from "CHIR+RA" condition. **e**, Representative BF images of PE/STM/PFH organoids ("CHIR+BMP4+RA" condition) at D11 and D17 of differentiation, and representative IF staining of D30 PE/STM/PFH organoids. Scale bars, 100 µm. ns, not statistically significant.

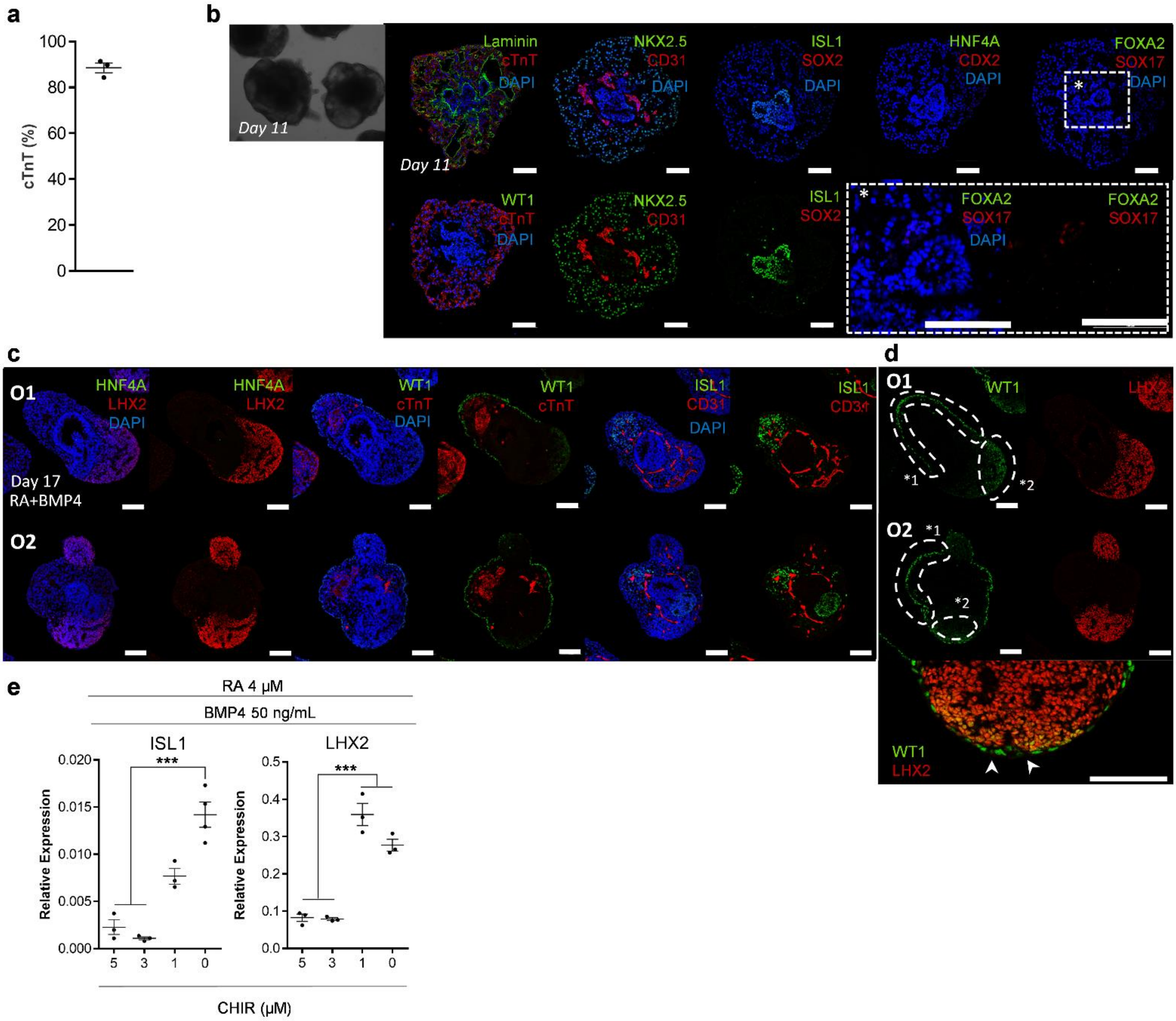

Supplementary Figure 6. Removal of WNT signaling activation during PE induction period opens the path for CM specification. (a-b), Flow cytometry analysis of the percentage of cTnT positive cells (Data are represented as mean ± SEM of n=3 independent experiments (a) and representative BF and IF images (b) of D11 aggregates from "BMP4" condition. **(c-d),** Representative IF image of D17 organoid obtained from "RA+BMP4" condition (c), and highlight of the WT1+/LHX2- cells that line the cTnT (regions *1) and LHX2 positive regions (white head arrow), but also WT1+/LHX2+ cells (regions *2) (d). Scale bars, 100 µm. **e**, Gene expression profile of *LHX2* and *ISL1* in PE induction condition with constant BMP4 (25 ng/mL) and RA (4 µM) concentration and decreasing concentration of CHIR. Values are normalized to *GAPDH*. Data are represented as mean ± SEM of n=3 independent experiments. Exact p-values: ISL1 – 3 vs 0 µM CHIR p=0.0004, 5 vs 0 µM CHIR p=0.0009; LHX2 – 3 vs 0 µM CHIR p=0.0003, 5 vs 0 µM CHIR p=0.0005. Ox nomenclature identifies different organoids obtained from at least 3 independent experiments. **$p < 0.01$, ***$p < 0.001$

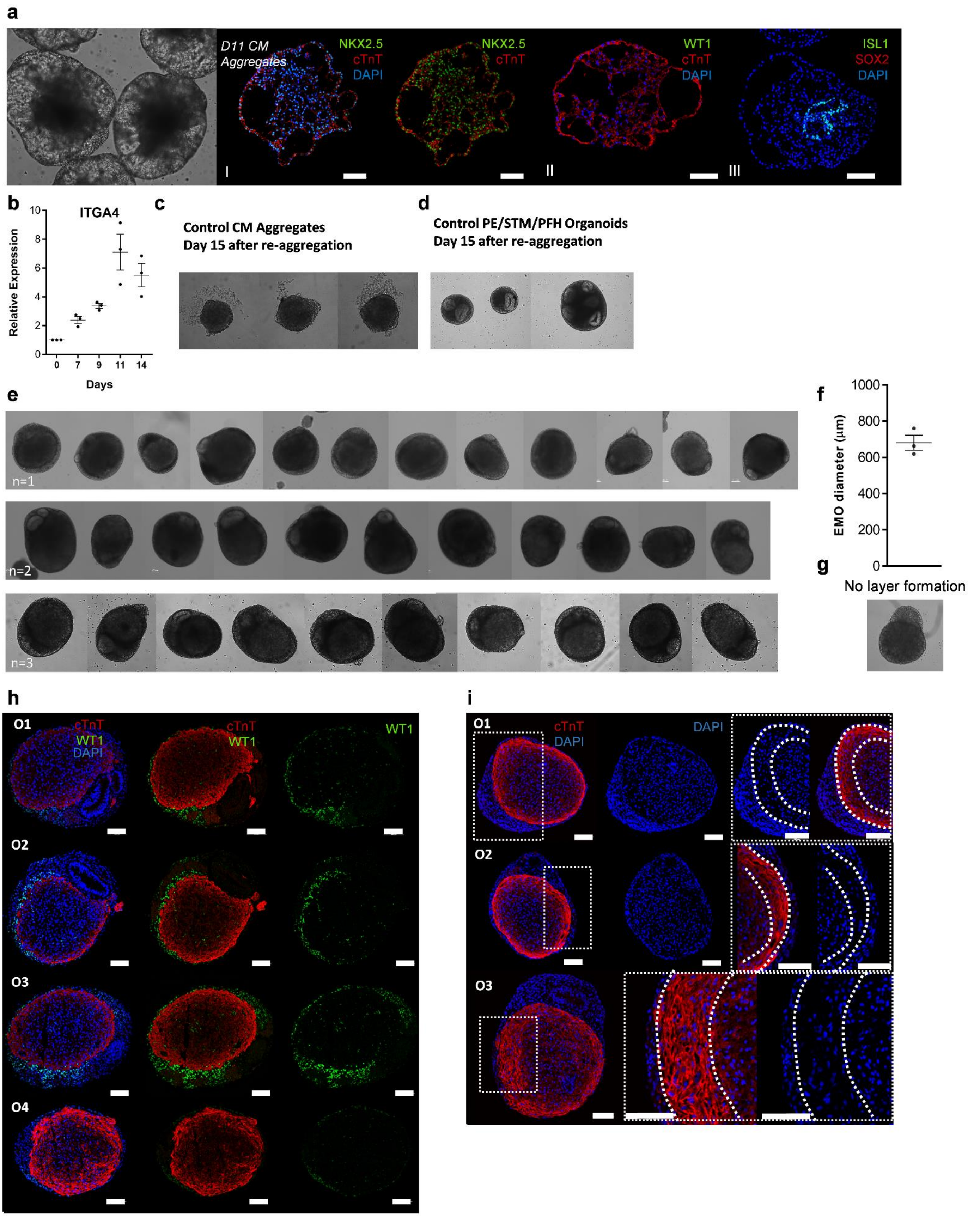

Supplementary Figure 7: Co-culture of PE/STM/PFH cells with hPSC-derived CM aggregates results in the development of a heart organoid showing a self-organized epicardial-like layer surrounding a myocardium-like tissue. a, Representative BF and IF staining of sections of CM aggregates at D11 of differentiation used for co-culture with PE/STM/PFH organoids. **b**, Gene expression profile of *ITGA4* in PE/STM/PFH organoids. Data are represented as

mean ± SEM of n=3 independent experiments. **c-d**, Representative BF images of control CM aggregates (c) and PE/STM/PFH organoids (d) 15 days post re-aggregation. **e**, Representative BF images of 10-12 EMOs after 15 days of co-culture for n=3 independent biological replicates. **f**, Distribution of EMOs diameter after 15 days of co-culture. Data are represented as mean ± SEM of n=3 independent experiments (n=1, 2 and 3 corresponds to the mean value of 10, 19, and 21 organoids, respectively). **g**, Representative BF image of unsuccessful EMOs organoids. **h-i**, Representative IF staining of EMOs sections after 15 days of co-culture, highlighting the epicardium- and myocardium-like layers (h) and the compact CM layer near the WT1 positive epicardial-like layer (i). Scale bars, 100 µm. Ox nomenclature identifies different organoids obtained from at least 3 independent experiments. Source data are provided as a Source Data file.

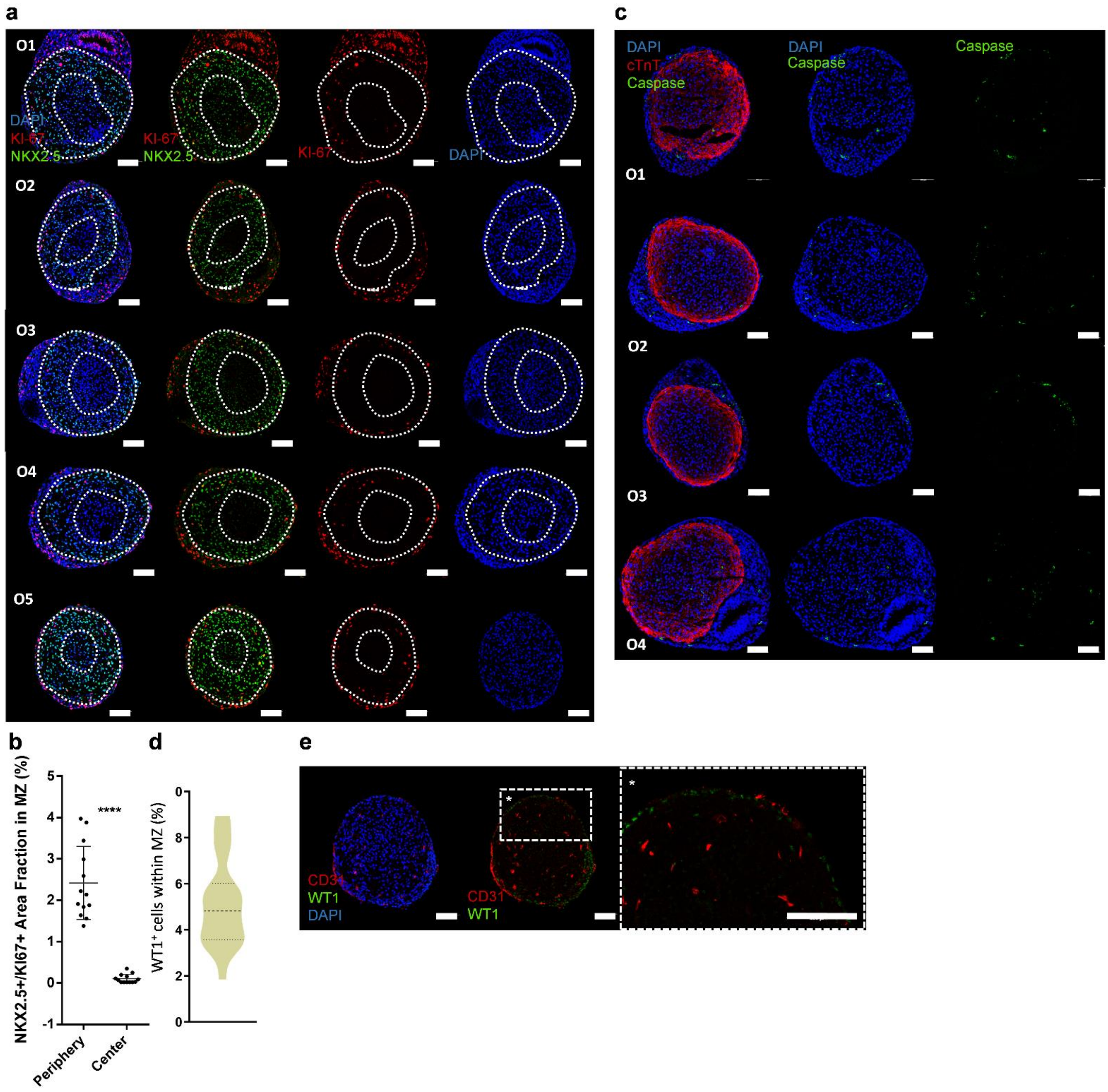

Supplementary Figure 8: EMOs present no signals of cell death and present improved signals of vascular and fibroblast cell composition compared with control CM aggregates. a, Representative IF staining of EMOs sections

after 15 days of co-culture, highlighting the prevalence of NKX2.5^{+}/Ki-67^{+} cells near the epicardial-like layer and the presence of NKX2.5$^{+low/-}$ towards the center of the organoid. Scale bars, 100 µm. O – Organoid. **b**, Quantification of NKX2.5/Ki-67^{+} cells in the periphery and center of EMOs in MZ. Data are presented as mean ± sd for 13 organoids from n=3 independent experiments. Exact p-value <0.0001. **c**, Representative IF staining of sections of EMOs sections after 15 days of co-culture highlighting the staining for the apoptotic marker caspase. Scale bars, 100 µm. **d**, Quantification of WT1 positive cells within the myocardium zone (MZ). **e**, Representative IF staining of EMOs sections after 15 days of co-culture, highlighting the CD31 positive cells. Scale bars, 100 µm. Ox nomenclature identifies different organoids obtained from at least 3 independent experiments. ****p < 0.0001. Source data are provided with this paper.

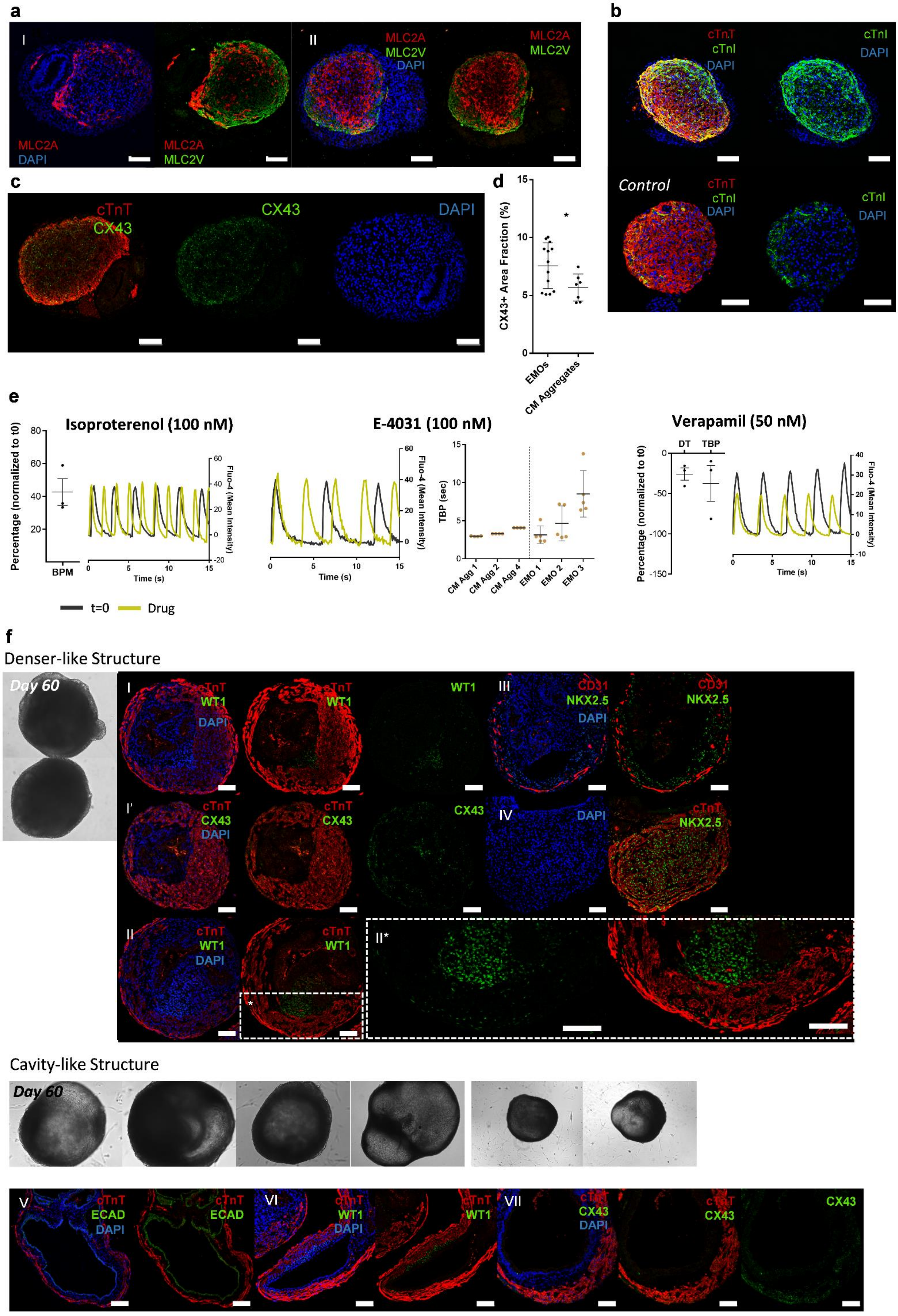
a
b
c
d
e
f
MLC2A
DAPI
MLC2V
cTnT
cTnI
Control
CX43
CX43+ Area Fraction (%)
EMOs
CM Aggregates
Isoproterenol (100 nM)
E-4031 (100 nM)
Verapamil (50 nM)
Percentage (normalized to t0)
BPM
Fluo-4 (Mean Intensity)
Time (s)
TBP (sec)
CM Agg 1
CM Agg 2
CM Agg 4
EMO 1
EMO 2
EMO 3
DT
TBP
t=0
Drug
Denser-like Structure
Day 60
WT1
CD31
NKX2.5
Cavity-like Structure
ECAD

Supplementary Figure 9: EMOs present improved signals of ventricle myocardium maturation compared with control CM aggregates. a-c, Representative IF staining of EMOs sections after 15 days of co-culture for the mature ventricle CM marker MLC2V (a), cTnI marker (b) and for connexin 43 (CX43) gap junction (c), highlighting the more intense staining of the mentioned markers near the edges of the EMO, which are in contact with the $WT1^+$ cells. Scale bars, 100 µm. **d**, Quantification of the percentage of area occupied by CX43 staining in MZ for EMOs and control CM aggregates. Data are presented as mean ± sd for n=13 organoids and n=7 CM aggregates from n=3 independent experiments. Exact p-value=0.0328. **e**, Calcium transient profiles of CM aggregates after 15 days of co-culture before and after drug stimulation with isoproterenol, verapamil and E-4031. Data are represented as mean ± SEM of n=3 independent experiments. BPM – Beats Per Minute; DT – Decay Time; TBP – Time Between Peak**. f**, Representative BF and IF images of EMOs after 60 days of co-culture, highlighting the two different types of structures observed, denser and cavity-like structure. Scale bars, 100 µm. *$p < 0.05$. Source data are provided with this paper.

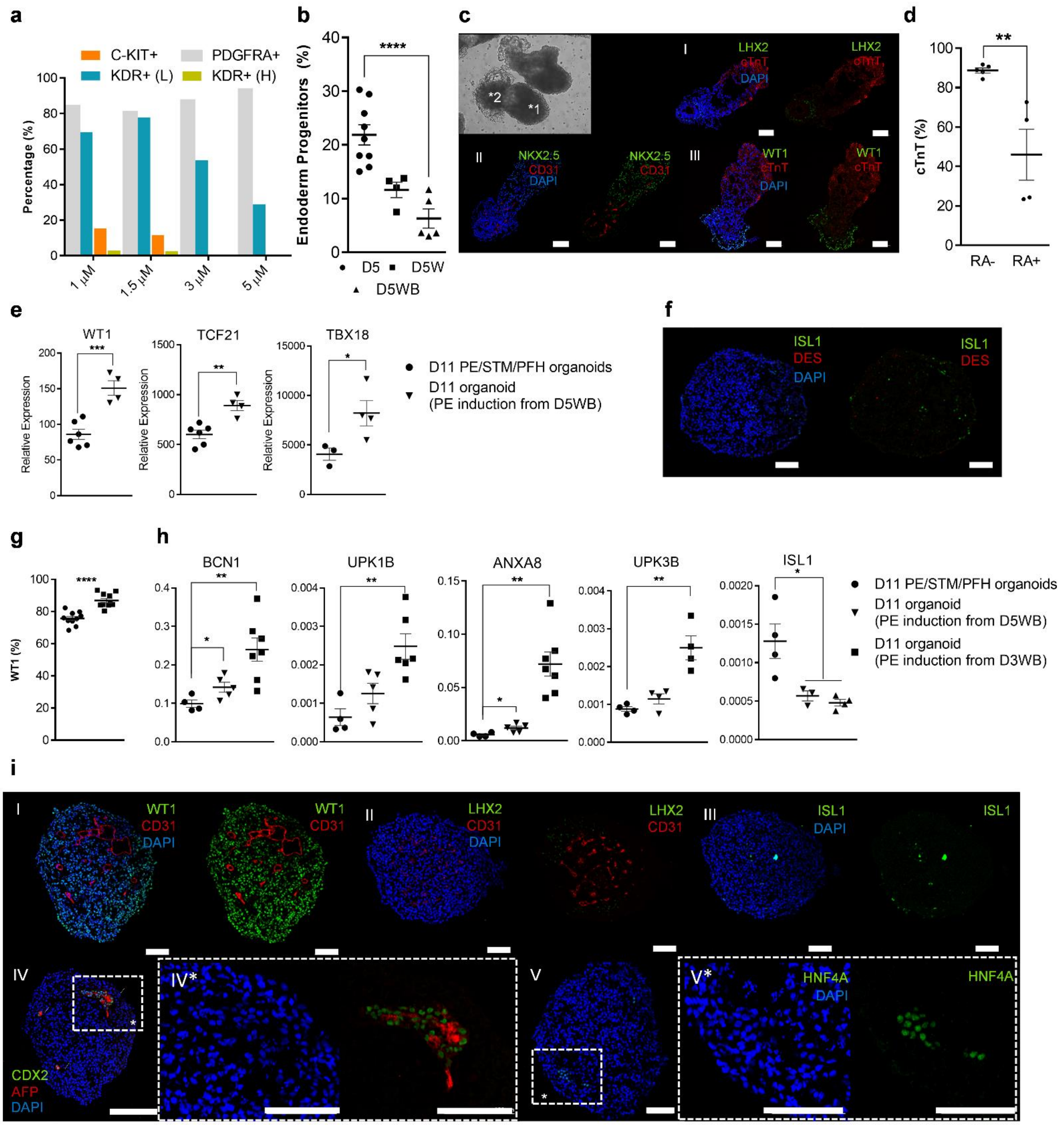

Supplementary Figure 10: Prolonged and combined activation of WNT and BMP signaling pathways during the first stages of hPSC differentiation conditioned CM and PE cells specification. a, Percentage of c-KIT, KDR and PDGFRA positive cells at D5 of differentiation, after WNT signaling activation using CHIR concentration from 1-5 µM. n=1 independent experiment. **b,** Percentage of c-KIT^{+}/KDR^{high-} cells at D5W and D5WB conditions compared with control D5. Data are represented as mean ± SEM of n=9 (D5), n=4 (D5W) and n=5 (D5WB) independent experiments. Exact p-value<0.0001. **c**, Representative BF and IF staining of D11 aggregates derived from CM differentiation protocol using D5WB progenitor cells. Aggregates show two distant areas, one exhibiting contraction (*1) and another that does not contract (*2). **d**, Percentage of cTnT positive cells at D11 of differentiation after RA treatment from D1-D13. Data are

represented as mean ± SEM of n=4 (RA+) and n=5 (RA-) independent experiments. Exact p-value=0.0072. **e**, Gene expression profile of *WT1*, *TBX18* and *TCF21* in organoids generated using PE induction from control and D5WB progenitor cells. Values are normalized to *GAPDH* and D0. Data are represented as mean ± SEM of n=3 (TBX18 D11 PE/STM/PFH), n=4 (TBX18, TCF21 and WT1 D11 PE induction D5WB) and n=6 (WT1 and TCF21 D11 PE/STM/PFH) independent experiments. Exact p-values: WT1 *p*=0.0007; TCF21 *p*=0.0022; TBX18 *p*=0.0489. **f**, Representative IF staining of D11 organoid sections obtained using PE induction from D5WB progenitor cells. **g**, Percentage of $WT1^+$ positive cells at D11 of differentiation using D3WB progenitor cell population for PE induction. Data are represented as mean ± SEM of n=10 independent experiments. Exact p-value<0.0001. **h**, Gene expression profile of *BCN1, UPK3B, UPK1B, ISL1* and *ANXA8* at D11 organoid obtained using PE induction from control D5, D3WB and D5WB progenitor cell populations. Values are normalized to *GAPDH*. Data are represented as mean ± SEM of n=3 (ISL1 PE induction D5WB), n=4 (BCN1, UPK1B, ANXA8, UPK3B and ISL1 D11 PE/STM/PFH, UPK3B PE induction D5WB, UPK3B and ISL1 PE induction D3WB), n=5 (BCN1, UPK1B and ANXA8 PE induction D5WB), n=6 (UPK1B PE induction D3WB) and n=7 (BCN1 and ANXA8 PE induction D3WB) independent experiments. Exact p-values: BCN1 D11 PE/STM/PFH vs PE induction D5WB *p*=0.0398, D11 PE/STM/PFH vs PE induction D3WB *p*=0.0084; UPK1B *p*=0.0033; ANXA8 D11 PE/STM/PFH vs PE induction D5WB *p*=0.0244, D11 PE/STM/PFH vs PE induction D3WB *p*=0.0019; UPK3B *p*=0.0023; ISL1 D11 PE/STM/PFH vs PE induction D5WB *p*=0.0463, D11 PE/STM/PFH vs PE induction D3WB *p*=0.0125. **i**, Representative IF staining of D11 organoid sections obtained using PE induction from D3WB progenitor cells. Scale bars, 100 µm.

Supplementary Table 1. List of antibodies used in Flow Cytometry and Immunostaining analysis.

Antibody	Source	Reference	Isotype	Dilution
**CD31**	Dako	M0823	Mouse IgG	1:50 (FC/IF)
**cTnT**	Thermo Fisher	MA5-12960	Mouse IgG	1:800 (FC)
**α-SMA**	Sigma-Aldrish	161208D	Rabbit IgG	1:200 (IF/FC)
**Calponin**	Abcam	ab700	Mouse IgG1	1:200 (FC/IF)
**NKX2.5**	Abcam	ab97355	Rabbit IgG	1:200 (IF)
**CXCR4**	Santa Cruz	Sc-12764	Mouse IgG2a	1:20 (FC)
**KDR-PE**	ReD Systems	FAB357P	Mouse IgG1	1:10 (FC)
**C-KIT-PE**	Biolegend	313204	Mouse IgG1	1:10 (FC)
**CD140a-APC**	BD Pharmingen	562777	Rat IgG2a	1:25 (FC)
**ISL1**	Abcam	Ab178400	Rabbit IgG	1:200 (FC/IF)
**WT1**	Abcam	ab89901	Rabbit IgG	1:100 (FC/IF)
**ALDH1A2**	Abcam	ab96060	Rabbit IgG	1:100 (IF)
**Collagen I**	Abcam	ab34710	Rabbit IgG	1:300 (IF)
**Laminin**	Abcam	ab11575	Rabbit IgG	1:300 (IF)
**Collagen IV**	Abcam	ab6311	Mouse IgG1	1:300 (IF)
**Fibronectin**	Abcam	ab253288	Mouse IgG1	1:300 (IF)
**Periostin**	Abcam	ab14041	Rabbit IgG	1:100 (IF)
**Vimentin**	Sigma	V6630	Mouse IgG1	1:300 (IF)
**E-Cadherin**	Cell Signaling Technology	24E10	Rabbit IgG	1:200 (IF)
**CDX2**	Abcam	ab76541	Rabbit IgG	1:200 (FC/IF)
**LHX2**	Abcam	ab184337	Rabbit IgG	1:200 (IF)
**HNF4A**	Santa Cruz Biotechnology	sc-374229	Mouse IgG1-488	1:50 (IF)
**AFP**	Merck/ Sigma-Aldrich®	A8452	Mouse IgG2a	1:500 (IF)
**DESMIN**	Santa Cruz	sc-271677	Mouse IgG2a	1:50 (IF)
**CD34**	Biolegend	343502	Mouse IgG1	1:25 (IF)
**SOX17**	Abcam	ab2224637	Mouse	1:200 (IF)
**SOX2**	ReD Systems	MAB2018	Mouse IgG2a	1:200 (IF)
**Ki-67**	Abcam	ab833	Rabbit IgG	1:50 (IS)
**CX43**	Sigma-Aldrish	C6219	Rabbit IgG	1:400 (IS)
**MLC2A**	Synaptic Systems	311011	Mouse IgG2b	1:200 (IS)
**MLC2V**	Proteintech	10906-1-AP	Rabbit IgG	1:200 (IS)
**CASPASE**	Cell Signaling	1679661S	Rabbit IgG	1:400 (IS)
**cTnI**	Abcam	ab47003	Rabbit IgG	1:100 (IS)
**Alexa Fluor 488 Donkey Anti-mouse IgG (H+L)**	Thermo Fisher	A21202	-	1:500 (IS)/1:1000 (FC)
**Alexa Fluor 488, Donkey Anti-rabbit IgG (H+L)**	Thermo Fisher	A21206	-	1:500 (IS)/1:1000 (FC)
**Alexa Fluor 546, Donkey Anti-mouse IgG (H+L)**	Thermo Fisher	A11030	-	1:500 (IS)/1:1000 (FC)
**Alexa Fluor 546, Donkey Anti-rabbit IgG (H+L)**	Thermo Fisher	A10040	-	1:500 (IS)/1:1000 (FC)

Supplementary Table 2. List of primers used in real time PCR analysis.

Gene	PRIMER (5´>3´)
**GAPDH**	FW: GAGTCAACGGATTTGGTCGT<br>RV: TTGATTTTGGAGGGATCTCG
**ISL1**	FW: GCGGAGTGTAATCAGTATTTGGA<br>RV: GCATTTGATCCCGTACAACCT
**NKX2.5**	FW: CCAAGGACCCTAGAGCCGAA<br>RV: GTCCGCCTCTGTCTTCTCCA
**TBX5**	FW: GAGGTGGGATAGTTGGAGAGC<br>RV: GAATCGCAGGGCAGGTCTTT
**TNNT2**	FW: GTCCAAACCAAAGCCCAGGT<br>RV: CCACTCTCTCTCCATCGGGG
**WT1**	FW: CAGCTTGAATGCATGACCTG<br>RV: TATTCTGTATTGGGCTCCGC
**TBX18**	FW: CCCAGGACTCCCTCCTATGT<br>RV: TAGGAACCCTGATGGGTCTG
**ALDH1A2**	FW: TCGCATCTTCGTGGAGGAGT<br>RV: TGCTCAGTGGTGGGGTCAAA
**TCF21**	FW: ACCCTCTTCCTCGCTTTCTC<br>RV: TGCTCTCGTTGGAAGTCACA
**PDGFRA**	FW: ATCGGAGGAGAAGTTTCCCAGAG<br>RV: GGTACTGCCAGCTCACTTCA
**ACTA2**	FW: CACTGTCAGGAATCCTGTGA<br>RV: CAAAGCCGGCCTTACAGA
**CNN1**	FW: GTCCACCCTCCTGGCTTT<br>RV: AAACTTGTTGGTGCCCATCT
**CD31**	FW: GCTGACCCTTCTGCTCTGTT<br>RV: TGAGAGGTGGTGCTGACATC
**MYH11**	FW: GGTCACGGTTGGGAAAGATGA<br>RV: GGGCAGGTGTTTATAGGGGTT
**GATA4**	FW: CTGAAGCTCTCCCCACAAGG<br>RV: GCTGTTCCAAGAGTCCTGCT
**UPK3B**	FW: CGGGTGAAGTTCCTCCTGATG<br>RV: GTCTTCCCTTGGTGGAGAGTG
**LHX2**	FW: CCACCACCCTGACAGACTTG<br>RV: AAGACGGACGTCACAGTTGG
**HLX1**	FW: TAGCCAGCCGAACACTTCTC<br>RV: GGGGCGAGTCAGAGAAACAG
**TBX1**	FW: TGTGGGACGAGTTCAACCAG<br>RV: GCTTCACTTGGAAGGTGGGA
**TBX20**	FW: GGGATGAGAGTCAGACAACCC<br>RV: AAGGCTGACCCTCGATTTGG
**BNC1**	FW: ACAGTGGAGGGCTGTAATGC<br>RV: ATGCTTCCTGGCTCAATGCT
**UPK1B**	FW: TGACCAACACAGCCTCTACC<br>RV: GCCCACAAATATGCCGATCC
**ANXA8**	FW: GCCTGCTCACTCCTCAGC<br>RV: CAGGATTTCCACCAGGCCAT
