# Supplementary material for: Human multilineage pro-epicardium/foregut organoids support the development of an epicardium/myocardium organoid
Source: Nat Commun. 2022 Nov 15;13:6981. doi: 10.1038/s41467-022-34730-7 (PMC9666429; doi:10.1038/s41467-022-34730-7)
Supplement: Supplementary file 3 — Description of Additional Supplementary Files [file 41467_2022_34730_MOESM3_ESM.pdf]

Description of Additional Supplementary Files

File Name: Supplementary Data 1
Description: Differential expression analysis for D3WB vs D3 and D5WB vs D5 conditions
