# Supplementary material for: Human multilineage pro-epicardium/foregut organoids support the development of an epicardium/myocardium organoid
Source: Nat Commun. 2022 Nov 15;13:6981. doi: 10.1038/s41467-022-34730-7 (PMC9666429; doi:10.1038/s41467-022-34730-7)
Supplement: Supplementary file 5 — Reporting Summary [file 41467_2022_34730_MOESM5_ESM.pdf]

nature portfolio

Corresponding author(s): Maria Margarida Diogo

Last updated by author(s): Oct 31, 2022

Reporting Summary

Nature Portfolio wishes to improve the reproducibility of the work that we publish. This form provides structure for consistency and transparency in reporting. For further information on Nature Portfolio policies, see our Editorial Policies and the Editorial Policy Checklist.

Statistics

For all statistical analyses, confirm that the following items are present in the figure legend, table legend, main text, or Methods section.

n/a	Confirmed	
☐	☒	The exact sample size (*n*) for each experimental group/condition, given as a discrete number and unit of measurement
☐	☒	A statement on whether measurements were taken from distinct samples or whether the same sample was measured repeatedly
☐	☒	The statistical test(s) used AND whether they are one- or two-sided<br>*Only common tests should be described solely by name; describe more complex techniques in the Methods section.*
☒	☐	A description of all covariates tested
☒	☐	A description of any assumptions or corrections, such as tests of normality and adjustment for multiple comparisons
☐	☒	A full description of the statistical parameters including central tendency (e.g. means) or other basic estimates (e.g. regression coefficient) AND variation (e.g. standard deviation) or associated estimates of uncertainty (e.g. confidence intervals)
☐	☒	For null hypothesis testing, the test statistic (e.g. *F*, *t*, *r*) with confidence intervals, effect sizes, degrees of freedom and *P* value noted<br>*Give P values as exact values whenever suitable.*
☒	☐	For Bayesian analysis, information on the choice of priors and Markov chain Monte Carlo settings
☒	☐	For hierarchical and complex designs, identification of the appropriate level for tests and full reporting of outcomes
☒	☐	Estimates of effect sizes (e.g. Cohen's *d*, Pearson's *r*), indicating how they were calculated

Our web collection on statistics for biologists contains articles on many of the points above.

Software and code

Policy information about availability of computer code

Data collection	Microsoft Excel 365; Graphpad Prism 6; R 4.1.2
Data analysis	For flow cytometry analysis: FlowJo V10; For immunofluorescence and BF images analysis: ImageJ 1.53c and Zen 2.3 (Blue Edition); For RNAseq data analysis: R 4.1.2; DESeq2 V1.32, STRING V11.5 (https://string-db.org/); For statistics analysis: Graphpad Prism 6; For RT-PCR analysis: StepOne Software v2.3

For manuscripts utilizing custom algorithms or software that are central to the research but not yet described in published literature, software must be made available to editors and reviewers. We strongly encourage code deposition in a community repository (e.g. GitHub). See the Nature Portfolio guidelines for submitting code & software for further information.

Data

Policy information about availability of data

All manuscripts must include a data availability statement. This statement should provide the following information, where applicable:

- Accession codes, unique identifiers, or web links for publicly available datasets
- A description of any restrictions on data availability
- For clinical datasets or third party data, please ensure that the statement adheres to our policy

The mesendoderm progenitor cell populations RNA-Sequencing data set generated in this study has been deposited in the National Center for Biotechnology Information Gene Expression Omnibus repository under accession code GSE184302 (https://www.ncbi.nlm.nih.gov/geo/query/acc.cgi?acc=GSE184302). The raw data generated in this study are provided in the Source Data file.

Field-specific reporting

Please select the one below that is the best fit for your research. If you are not sure, read the appropriate sections before making your selection.

☒ Life sciences ☐ Behavioural & social sciences ☐ Ecological, evolutionary & environmental sciences

For a reference copy of the document with all sections, see nature.com/documents/nr-reporting-summary-flat.pdf

Life sciences study design

All studies must disclose on these points even when the disclosure is negative.

Sample size	For all experiments, n=3-12 independent biological replicates were used in accordance with common practice in this field (Drakhlis et al. 2021; Lewis-Israeli et al. 2021). Each experiment comprise more than 100 organoids.
Data exclusions	No data were excluded.
Replication	Replications are mentioned in figure legends. Most experiments represent n=3-12 independent biological experiments for flow cytometry and PCR data. Immunofluorescence images are representative of n=3 independent biological replicates and 10-30 organoids were collected from each experiment. Additional cell lines were used to prove the reproducibility of the presented data. For RNAseq data n=3 biological independent replicates were analyzed for each condition.
Randomization	For each sample a pool of organoids/aggregates were randomly collected from a n>100 population of organoids/aggregates that were cultured in 6 ULA-well plates.
Blinding	The generation of PE/STM/PFH, EMOs organoids and CM aggregates from hPSC lines using different culture conditions was performed in a non-blinded manner, and consistent and defined quality criteria were defined to avoid investigator bias selection for further analysis. Image acquisition, image quantification and flow cytometry analysis was performed with consistent parameters to avoid exclusion of data due to investigator bias. Calcium transient acquisition and analysis was performed in a blinded manner.

Reporting for specific materials, systems and methods

We require information from authors about some types of materials, experimental systems and methods used in many studies. Here, indicate whether each material, system or method listed is relevant to your study. If you are not sure if a list item applies to your research, read the appropriate section before selecting a response.

Materials & experimental systems

n/a	Involved in the study	
☐	☒	Antibodies
☐	☒	Eukaryotic cell lines
☒	☐	Palaeontology and archaeology
☒	☐	Animals and other organisms
☒	☐	Human research participants
☒	☐	Clinical data
☒	☐	Dual use research of concern

Methods

n/a	Involved in the study	
☒	☐	ChIP-seq
☐	☒	Flow cytometry
☒	☐	MRI-based neuroimaging

Antibodies

Antibodies used

cTnI, Abcam (ab47003)
MLC2A, Synaptic Systems (311011)
MLC2V, Proteintech (10906-1-AP)
Caspase-3, Cell Signaling (1679661S)
CD140a-APC, BD Pharmingen (562777)
ISL1 Abcam (ab178400)
WT1 Abcam (ab89901)
ALDH1A2 Abcam (ab96060)
Collagen, type I, Abcam (ab34710)
Laminin, Abcam (ab11575)
Collagen, type IV, Abcam (ab6311)
Fibronectin, Abcam (ab253288)
Periostin, Abcam (ab14041)
Vimentin, Sigma-Aldrich (V6630)
E-Cadherin, Cell Signaling Technology (24E10)
CDX2, Abcam (ab76541)
LHX2, Abcam (ab184337)

HNF4A-Alexa 488, Santa Cruz Biotechnology (sc-374229)
AFP, Sigma-Aldrich (A8452)
DESMIN, Santa Cruz Biotechnology (sc-271677)
CD34, Biolegend (343502)
SOX17, Abcam (ab224637)
SOX2, ReD Systems (MAB2018)
Ki-67, Abcam (ab833)
CX43, Sigma-Aldrich (C6219)
CD31, Dako (M0823)
cTnT, Thermo Fisher Scientific (MA5-12960)
α-SMA, Sigma-Aldrich (161208D)
Calponin, Abcam (ab700)
NKX2.5, Abcam (ab97355)
CXCR4, Santa Cruz Biotechnology (Sc-12764)
KDR-PE, ReD Systems (FAB357P)
c-KIT-PE, Biolegend (313204)
Alexa Fluor 488, Donkey Anti-mouse IgG (H+L), Thermo Fisher Scientific (A21202)
Alexa Fluor 488, Donkey Anti-rabbit IgG (H+L), Thermo Fisher Scientific (A21206)
Alexa Fluor 546, Donkey Anti-mouse IgG (H+L), Thermo Fisher Scientific (A11030)
Alexa Fluor 546, Donkey Anti-rabbit IgG (H+L), Thermo Fisher Scientific (A10040)

Validation

MLC2A: Suitable for WB, IHC-P, ICC/IF. Validated for detection of human MLC2A protein
MLC2V: Suitable for WB, IP, IHC, IF, ELISA. Validated for detection of human MLC2V protein
Caspase-3: Suitable for WB, IP, IHC, IF. Validated for detection of human Caspase protein
CD140a-APC: Suitable for FC. Validated for detecting cell surface marker CD140 subunit alpha in humans
ISL1: Suitable for FC, IF, IHC-P, WB. Validated for detecting ISL1 in human tissue
WT1: Suitable for WB, IHC-P, Flow Cyt (Intra), ICC/IF. Validated for detection of human WT1 protein
ALDH1A2: Suitable for IF, WB, IHC-P. Validated for detection of human ALDH1A2 protein
Collagen, type I: Suitable for IF, WB. Validated for detection of human collagen type I
Laminin: Suitable for IF, IHC-P. Validated for detection of human laminin
Collagen, type IV: Suitable for IF, IHC-P. Validated for detection of human collagen type IV
Fibronectin: Suitable for ELISA, ICC/IF, WB. Validated for detection of human fibronectin
Periostin: Suitable for ELISA, ICC/IF, IHC-Fr, IHC-P, WB. Validated for detection of human periostin protein
Vimentin: Suitable for IF, IHC-P, WB. Validated for detection of human vimentin
E-Cadherin: Suitable for WB, IP, IHC, IF, FC, ELISA. Validated for the detection of E-cadherin in human tissue
CDX2: Suitable for FC, WB, ICC/IF, IHC-P. Validated for detection of human, mouse and rat CDX2 protein
LHX2: Suitable for IF, WB, IHC-Fr, IP, IHC-P. Validated for detection of human, mouse and rat LHX2 protein
HNF4A-Alexa 488: Suitable for IF, IP; HRP for WB, IHC(P) and ELISA. Validated for detection of human HNF4 alpha
AFP: Suitable for IF, IHC, ELISA. Validated for detection of AFP protein in human fetal liver
DESMIN: Suitable for IF, WB, IF, IHC, ELISA. Validated for detection of human and mouse desmin
CD34: Suitable for IF, IHC, FC. Validated for detection of the cell surface marker CD34 in human and mouse tissue
SOX17: Suitable for IF, IHC-P, IP, ICC, WB. Validated for detection of SOX17 in human cells
SOX2: Suitable for IF, WB, IHC, FC. Validated for detection of human and mouse SOX2
Ki-67: Suitable for IHC-P, IF. Validated for detection of Ki-67 in human samples
CX43: Suitable for IF, WB, IHC. Validated for detection of CX43 in heart human samples
CD31: Suitable for IF, IHC. Validated for detection of the cell surface marker CD31 in human tissue
cTnT: Suitable for FC, IHC, IF, WB. Validated for detection of cardiac troponin T in human cardiac muscle
α-SMA: Suitable for IF, IHC, WB. Validated for detection of smooth muscle actin in human cardiac muscle
Calponin: Suitable for FC, ICC/IF, IHC-FoFr, IHC-P, IHC-Fr. Validated for detection of calponin in human tissue
NKX2.5: Suitable for IHC-P, WB, ICC/IF. Validated for detection of NKX2.5 in human cardiac muscle
CXCR4: Suitable for FC. Validated for detection of the cell surface marker CXCR4 in human samples
KDR-PE: Suitable for FC. Validated for detection of the cell surface marker KDR in human samples
c-KIT-PE: Suitable for FC. Validated for detection of the cell surface marker c-KIT in human samples

Eukaryotic cell lines

Policy information about cell lines

Cell line source(s)

All cell lines were purchased to commercial companies, except H9 (WiCell, WA09) which was a gift from Inês Milagre's Lab (IGC-Instituto Gulbenkian de Ciência)
The iPS-DF6-9-9T.B cell line was purchased from WiCell (iPS-DF6-9-9T)
The F002.1A.13 was from purchased Tecnologias Celulares para Aplicação Médica, Unipessoal, Lda

Authentication

Purchased cells were authenticated by vendors, typically by STR profiling.

Mycoplasma contamination

Mycoplasma testing was performed routinely in our lab and all the cell lines tested negative

Commonly misidentified lines
(See ICLAC register)

No commonly misidentified lines were used

Flow Cytometry

Plots

Confirm that:

☒ The axis labels state the marker and fluorochrome used (e.g. CD4-FITC).

☒ The axis scales are clearly visible. Include numbers along axes only for bottom left plot of group (a 'group' is an analysis of identical markers).

☒ All plots are contour plots with outliers or pseudocolor plots.

☒ A numerical value for number of cells or percentage (with statistics) is provided.

Methodology

Sample preparation	hPSC-derived organoids were dissociated using trypsin-EDTA 0.25% for 7 minutes and fixed using PFA2% for 30 minutes
Instrument	FACs Calibur (Becton Dickinson)
Software	FlowJo Software
Cell population abundance	A total of 10000 events were collected for each sample
Gating strategy	For each analyzed condition, a control sample comprising only the secondary antibody was used to identify the negative population and to establish the boundary for the positive gate, which included a maximum of 1% of the negative population.

☒ Tick this box to confirm that a figure exemplifying the gating strategy is provided in the Supplementary Information.

March 2021
